# Factors influencing acceptance and trust of chatbots in juvenile offenders’ risk assessment training

**DOI:** 10.3389/fpsyg.2023.1184016

**Published:** 2023-06-16

**Authors:** Ann-Pierre Raiche, Léanne Dauphinais, Manon Duval, Gino De Luca, David Rivest-Hénault, Thomas Vaughan, Catherine Proulx, Jean-Pierre Guay

**Affiliations:** ^1^School of Criminology, University of Montreal, Montreal, QC, Canada; ^2^National Research Council Canada, Boucherville, QC, Canada

**Keywords:** virtual learning-based simulation, chatbot, education, training, risk assessment, acceptance, trust

## Abstract

**Introduction:**

Research has identified simulation-based training with chatbots and virtual avatars as an effective educational strategy in some domains, such as medicine and mental health disciplines. Several studies on interactive systems have also suggested that user experience is decisive for adoption. As interest increases, it becomes important to examine the factors influencing user acceptance and trust in simulation-based training systems, and to validate applicability to specific learning tasks. The aim of this research is twofold: (1) to examine the perceived acceptance and trust in a risk assessment training chatbot developed to help students assess risk and needs of juvenile offenders, and (2) to examine the factors influencing students’ perceptions of acceptance and trust.

**Methods:**

Participants were 112 criminology students in an undergraduate course in a Canadian university. Participants were directed to use a custom-designed chatbot with a virtual 3D avatar for juvenile offenders’ risk assessment training, to complete online questionnaires and a risk assessment exercise.

**Results:**

Results show satisfactory levels of acceptance and trust in the chatbot. Concerning acceptance, more than half appeared to be satisfied or very satisfied with the chatbot, while most participants appeared to be neutral or satisfied with the benevolence and credibility of the chatbot.

**Discussion:**

Results suggest that acceptance and trust do not only depend on the design of the chatbot software, but also on the characteristics of the user, and most prominently on self-efficacy, state anxiety, learning styles and neuroticism personality traits. As trust and acceptance play a vital role in determining technology success, these results are encouraging.

## Introduction

### Education and simulation

The didactic lecture format is the dominant teaching method within most higher education courses across disciplines (Butler, 1992). This method is commonly used because of its economical and practical features, especially with many students and limited resources (Alaagib et al., 2019). Although didactic lecture is one of the most common teaching methods, it presents numerous limitations across disciplines, especially those requiring clinical learning and training skills. For example, the concepts taught during didactic lectures are difficult to translate into practice and opportunities to gain clinical experience with real patients are limited (Mazmanian and Davis, 2002; Rizzo and Talbot, 2016). Research has shown that to be more effective, those lectures must be used combined with other methods and techniques (Butler, 1992; Meyers and Jones, 1993).

Simulations-based learning is considered one of the most effective methods to improve the learning of complex skills across disciplines (Chernikova et al., 2020). Simulation is defined as “[…] a technique (not a technology) to replace and amplify real experiences with guided ones, often ‘immersive’ in nature, that evoke or replicate substantial aspects of the real world in a fully interactive fashion” (Lateef, 2010, p. 348). Simulations can range in complexity and presentation, for example peer-to-peer role play or using live actors to portray patients (Chernikova et al., 2020). Currently, training methods using simulated patients are considered the gold standard to develop interviewing, assessment, and diagnostic skills in nursing, medicine, and psychology (Mooradian, 2008; McGaghie et al., 2011).

Simulations can also be enhanced by technology. Cook et al. (2013) define technology-enhanced simulation as an “educational tool or device with which the learner physically interacts to mimic real life and in which they emphasize the necessity of interacting with authentic objects” (p. 876). Technology-enhanced simulation offers innovative solutions to address many limitations associated with the use of standard simulated patients (Washburn et al., 2016). Until recently, there were very few avenues available to organizations wishing to enhance the knowledge of students, but technological advances have enabled the development of innovative methods.

### Artificial intelligence and education

There is a growing interest in the use of artificial intelligence in the field of education (Roos, 2018; Okonkwo and Ade-Ibijola, 2021). To support teaching and learning activities, chatbots powered by artificial intelligence are one of the most popular technology-enhanced simulation applications across fields of study such as nursing, medicine, psychology (Okonkwo and Ade-Ibijola, 2021). In the fields of clinical psychology, psychiatry, social work, criminology and particularly in learning tasks, various chatbots have been created in the last years. For example, in the medical field, Kenny et al. (2007) developed “Justin,” a human virtual agent used to practice professional interviewing techniques as well as to improve recognition of signs and symptoms of behavioral disorders. More recently, Washburn et al. (2016, 2020), developed six different virtual personas designed to allow social work students to practice asking interview questions, creating a positive therapeutic alliance, and gathering clinical information to recognize mental health disorders.

In artificial intelligence research, terms like chatbot, conversational agent, embodied conversational agent, virtual agent, virtual assistant, and even avatar are used synonymously and interchangeably (von der Pütten et al., 2010). Although there are some subtle distinctions between these terms (see McTear, 2020 for more details), for the purpose of this study, the term chatbot is used, and refers to

… digital tools existing either as hardware (such as an Amazon Echo running the Alexa digital assistant software) or software (such as Google Assistant running on Android devices or Siri running on Apple devices) that use machine learning and artificial intelligence methods to mimic humanlike behaviors and provide a task-oriented framework with evolving dialogue able to participate in conversation (Vaidyam et al., 2019, p. 457).

There are two categories of chatbots, “simple chatbot” and “smart or advanced chatbot” (Veretskaya, 2017). Simple chatbots are rule-based chatbots, which means that they depend on prewritten keywords chosen by the developer. In other words, predetermined options restrict user interaction and there are very few opportunities for free responses from the user. For example, if a user enters a question without one of the prewritten keywords, the chatbot won’t be able to understand the question and will respond a default message like “Sorry, I did not understand” (Veretskaya, 2017). Despite these restrictions, simple chatbots are widely used in several areas because they are easy to use and quick to implement (Schmitt, n.d.). Smart or advanced chatbots are artificial intelligence-based chatbots, which means they use Machine learning (ML) and Natural Language Processing (NLP). ML is a “branch of artificial intelligence and computer science which focuses on the use of data and algorithms to imitate the way that humans learn” (IBM Cloud Education, 2020a, para 1), while NLP refers to “the branch of computer science—and more specifically, the branch of artificial intelligence or AI—concerned with giving computers the ability to understand text and spoken words in much the same way human beings can” (IBM Cloud Education, 2020b, para 1).

Virtual simulated-based learning using chatbot systems present several advantages over traditional learning methods. One of the benefits is the great versatility and adaptability of the virtual characters. Chatbots offer the possibility to create diverse personalities or case studies with different physical/sociodemographic characteristics such as hair color, skin color, gender, and age, but also different clinical needs such as mental health concerns, physical health concerns, criminal dynamics, etc. (Washburn et al., 2020). Another advantage is availability and accessibility. Chatbots can be installed on or accessed from personal computers and do not require a specific space or specialized equipment. Effectively, they can be used at any moment and at any place (Triola et al., 2006; Washburn and Zhou, 2018). They can also be used repeatedly and by multiple users at the same time, which can be particularly useful for large cohorts of students (Washburn and Zhou, 2018; Washburn et al., 2020). In addition, unlike traditional approaches using actors, systems using chatbots are not subject to the variability within actors or the availability issues of actors, stakeholders, and organizations (Washburn et al., 2020). In the long run, the use of virtual patients may be more affordable than actor-based simulations as they can be used yearly and can be shared across departments or institutions (Washburn et al., 2020). Chatbot programs are not only a safe learning environment for students but also for patients or clients. They offer students a space to safely try new approaches and new techniques. As many professionals from different fields such as medicine or psychology work with vulnerable populations, it is important to offer students a place where they can make mistakes and try strategies without having a negative impact on their patients (Kenny et al., 2008; Washburn and Zhou, 2018; Coyne et al., 2021). Chatbots can also offer systematic feedback to the user. Some chatbot programs automatically save a text log of their interactions with their user, which can be used to review their performance, including successes and mistakes (Washburn et al., 2020).

Research on virtual simulated-based learning using chatbot systems identifies this method as an effective educational strategy (Chernikova et al., 2020). Research suggests that the skills learned by students using virtual patient simulations can be equivalent to the skills learned using standard simulations with actors (Cook et al., 2010) and that these skills are applicable in real-world situations involving patients (Triola et al., 2006; Washburn et al., 2016). Previous studies have focused on mechanisms that explain the effectiveness of this educational strategy. Those studies suggest that factors such as interactivity, ease of use, well-developed backstories, the realism of the clinical scenarios, and the availability of timely feedback increased usability and clinical skill acquisition (Cook et al., 2010; Bateman et al., 2012).

### Factors influencing acceptance and trust

As interest in chatbots as an effective learning tool increase, it is important to examine the factors that influence user acceptance and trust to use them. In their systematic review, Ling et al. (2021) have identified five categories of factors that influence chatbot adoption, namely usage-related factors (such as perceived usefulness and ease of use), agent-related factors (such as visual appearance and gesturing), user-related factors (such as demographic information and technology experience), attitude and evaluation factors (such as attitudes and satisfaction), and other factors (such as social influence). This study focuses on the user-related factors because studies suggest that these factors can influence engagement, acceptance, and trust in technologies but that they have not been sufficiently studied (Philip et al., 2020).

#### Acceptance

Several factors were identified to impact acceptance of chatbot. As present by Ling et al. (2021), these factors included demographic factors (gender, age), users’ expertise with technology and psychological factors.

Some studies indicate that there are some age-related differences in the usability and acceptance of a chatbot. Research in the field of technology acceptance indicates that perceived ease of use and perceived security of several technologies differ between older and younger adults (Grimes et al., 2010; Mitzner et al., 2010). Grimes et al. (2010) found out that older adults are less likely to be using technologies and less knowledgeable about security than younger adults (Grimes et al., 2010). Other research suggests that there is no difference between age groups and that the relation between age and technology acceptance is a complex one (Mitzner et al., 2010; McLean and Osei-Frimpong, 2019).

Research conducted more than a decade ago also suggested gender-related differences (Thompson and Lim, 1996; Milis et al., 2008; Padilla-Meléndez et al., 2008). There are some gender differences in perceptions of whether the technologies are easy to use. Thus, females tend to view technologies as being less easy to use compared to males (Thompson and Lim, 1996; van Braak, 2004; Milis et al., 2008). The results also show that males appear to have more previous experience with technologies than females (Thompson and Lim, 1996). Moreover, more recent research about technology acceptance indicated the opposite. Milis et al. (2008) suggest that females feel insecure when using a new virtual learning environment due to the novelty. However, they also indicate that females with attitudes more favorable toward thinking and learning are more likely to have a more favorable perception of usability. In opposite, males feel more secure, but they need an external motivation to engage in a virtual learning environment. In their study about the acceptability of an application for collecting symptom and quality-of-life information for patients, Wolpin et al. (2008) found that women found the program more acceptable than man. There is also inconsistency within research regarding the difference between males and females. Although some studies suggest that gender plays a significant role in determining the intention of accepting new technology, other studies found no differences between males and females (Suri and Sharma, 2013; McLean and Osei-Frimpong, 2019).

Beyond the degree of experience or familiarity with technology, research suggests that the user’s immersive tendencies can influence chatbot acceptance. Previous research demonstrates that participants with highly immersive tendencies will feel more present in the virtual environment and enjoy the experience more than a participant who does not generally become immersed in activities (Witmer and Singer, 1998; Johns et al., 2000; Nunez, 2003).

In terms of personality traits, their effects on technology acceptance have rarely been studied. Available research shows that different personality traits impact acceptance (McKnight et al., 2002; Brown et al., 2004; Müller et al., 2019). Research demonstrates that curiosity (Brandtzaeg and Følstad, 2017), personal innovativeness (Frambach et al., 2000; Richad et al., 2019), and hypervigilance (Mäurer and Weihe, 2015) have a positive influence on their perception of acceptance and usefulness of chatbots. In addition, research suggests that openness to experience and extraversion are also positively related to the acceptance of new technology (Islam et al., 2017). Research also suggests that self-efficacy and anxiety can play a role in technology acceptance (Czaja et al., 2006). In their study, Czaja et al. (2006) found that computer self-efficacy was an important predictor of general use of technology and that people with lower self-efficacy are less likely to use technology in general. They also found that self-efficacy has an indirect effect on technology adoption through anxiety, such that people with lower self-efficacy would have higher anxiety.

In addition, psychological traits such as learning styles seem to play a role in explaining and understanding user reactions to systems. Learning styles refer to the preferential way in which the individual absorbs, processes, and retains information and skills (Reid, 1995). Individual learning styles depend on cognitive, affective, environmental factors, and prior experience (Othman and Amiruddin, 2010). Studies on learning styles suggests that it is important to match the learning and teaching styles because it affects academic achievement and learner satisfaction (Felder and Silverman, 1988; Felder, 1993; Coffield et al., 2004). However, some others suggest that mismatch (i.e., using teaching style that are not suitable with learning style) might challenge students to adjust and learn in more integrated ways (Entwistle, 1988; Robotham, 1995; Vita, 2001). Despite some inconsistencies in the studies about the relationship between learning style and technology acceptance, the relationship between learning styles and perceived satisfaction is evident (Felder and Brent, 2005). Within the psychological domain, some authors claim that the learning style is one of the most important individual differences that affect learner performance and satisfaction, which also influences acceptance (Dunn and Dunn, 1974; Felder, 1988; Kolb and Kolb, 2005). According to these authors, learning styles can motivate students and thereby enhance sense of achievement and/or satisfaction.

#### Trust

Concerning trust, few studies have focused on factors identified to impact trust of chatbot. These studies also indicate that there are some age-related differences in the trust of a chatbot. Hoff and Bashir (2015) suggested that older people trust automated processes less than younger people. Følstad and Brandtzaeg (2020) also found out that older adults appreciated the pragmatic chatbot attributes (i.e., usefulness and usability) while younger participants appreciated the hedonic chatbot attributes (i.e., characteristics associated with the mental or emotional wellbeing of the user).

#### Acceptance, trust in chatbot and education

Studies on interactive systems emphasize on the fact that acceptance and trust play a vital role in determining technology success. User experience is decisive for the adoption and implementation of such systems, especially in education (Young et al., 2008; Hornbæk and Hertzum, 2017). When accepted and implemented correctly, chatbots can be a useful technology to facilitate learning within the educational context (Clarizia et al., 2018). Until now, very few studies have looked at the user-related factors that influence acceptance and trust of a chatbot in a training context. Indeed, except for demographic factors such as age and gender, knowledge is very limited.

In health-related professions, the level of education and clinical competency is a key factor in improving client outcomes (Coyne et al., 2021). Professionals must be competent in interviewing techniques, symptom/ability assessment, diagnosis, motivational interviewing, and interpersonal communication. An effective interview structure needs to cover all areas of potential clinical concerns and no mistakes can be made (Fernández-Ballesteros et al., 2003). In the course of their work, professionals are asked to interact and make crucial decisions in sensitive contexts that may have an influence on both individuals being assessed and on society. In the forensic field, it is the responsibility of the professionals to assess the risk of violence. Risk assessment is a process involving the systematic collection of information from several sources (e.g., data collection from interviews, case files, family, parents, employers, or teachers) to determine whether someone is likely to use violence, against themselves or another person, in the near future. This evaluation is important since it allows professionals to establish a treatment plan adapted to the person’s needs, treatment plan which aim to reduce the risk of violence and promote community reintegration (Guay et al., 2022). To do this evaluation, professionals use structured risk assessment instruments. For both adults and youth in Canada, these assessments are conducted systematically and influence the entire judicial process, particularly at the release level. It is crucial for public safety that professionals are competent because a bad decision can have serious impacts on public safety.

## The current research

To our knowledge, there is a limited number of chatbots with virtual avatars available that are useful for training professionals working in the forensic field. This research aims to examine how are acceptance and trust perceived in a recently developed juvenile risk assessment training chatbot, and what are the user-related factors influencing this perception? In this order, the aim of this research is twofold:

1. Examine the perceived acceptance and trust in a risk assessment training chatbot developed to assess risk and needs of juvenile offenders.
2. Examine the factors influencing students’ perception of acceptance and trust.

## Materials and methods

### Participants and recruitment

Participants were all criminology students at a Canadian university. More precisely, participants were mostly female, between 20 and 25 years old and in their second year of criminology program. Recruitment of participants took place from January 2022 to April 2022, in an undergraduate course on risk assessment. As part of the course and separately from this study, 112 students were asked to complete questionnaires and a scoring exercise based on an interview with a simulated offender (chatbot). At the end of the course, all students were verbally solicited by the professor. All students were informed that participation was independent of any class credit or grade, and consent was requested after the final grade was delivered to students. All interested participants gave their written informed consent before entering the study. Ethical approval from the University of Montreal (#CERSC-2022-024-D) and CÉR-Jeunes en difficulté (#MP-CER-JD-20-19) was obtained.

### Data collection procedures

Participants were invited to complete different online questionnaires and complete the risk assessment exercise using the chatbot. In addition, participants answered a series of open-ended questions about strengths, limitations, difficulties encountered, recommendations for improvement and benefits from the chatbot exercise. All data were collected with LimeSurvey (Limesurvey GmbH, 2003). The risk assessment tool used to complete the exercise with the chatbot is the Youth Level of Service/Case Management Inventory (YLS/CMI). The YLS/CMI is one of the most widely used structured risk and need assessment measures across many countries. The validity of the YLSC/CMI is supported by several peer-reviewed and published studies conducted with different research groups (Catchpole and Gretton, 2003; Schmidt et al., 2005; Onifade et al., 2008; Rennie and Dolan, 2010; McGrath and Thompson, 2012; Takahashi et al., 2013; Campbell et al., 2014; Chu et al., 2015). The YLS/CMI is a standardized instrument that estimates the level of risk of recidivism by assessing the number of static and dynamic recidivism risk factors present in the lives of young offenders aged 12–18 (Hoge and Andrews, 2011). The YLS/CMI assesses the presence or absence of 42 factors that have been grouped into eight domains empirically related to re-offending: Prior and Current Offenses, Family Circumstances/Parenting, Education/Employment, Peer Relations, Substance Abuse, Leisure/Recreation, Personality/Behavior and Attitudes/Orientation. The YLS/CMI is the preferred instrument in this study, as it is widely used in Quebec.

### The chatbot

#### Conversation engine

The chatbot software used in this study has been developed in collaboration with the National Research Council of Canada. The software is based on Rasa, an open-source framework, which leverages ML for building AI assistants and chatbots (Bocklisch et al., 2017). Rasa is based on two principles, namely Natural Language Understanding (NLU) and Dialogue Management. NLU (named Rasa NLU) extracts intents and entities from the user’s messages, while Dialogue Management (named Rasa Core) leverages stories and rules to determine what the bot will do or say based on the user’s message and context of the conversation (Bocklisch et al., 2017).

The chatbot software runs on a standard desktop or personal laptop computer. Communication with the chatbot can be done through voice leveraging a speech-to-text service, and via a text-based interface if necessary. In other words, participants would speak to the chatbot, then the user would review the text generated by the speech-to-text service before submitting it to the engine. The chatbot would answer vocally and with text. A text box of the conversation between the participant and the chatbot would also be generated for later feedback.

#### Chatbot development

The platform was developed with Unity 3D, a game development platform used to create and operate interactive, real-time 3D content (Unity Technologies, 2021). Character models were created with a universal framework called MakeHuman (MakeHuman Community, 2016). MakeHuman is an open-source tool for making 3D characters. The software offers more than 3,000 parameters to create highly detailed and unique characters: hair, skin, measurements, tooth shape, posture, etc.

**FIGURE 1:**
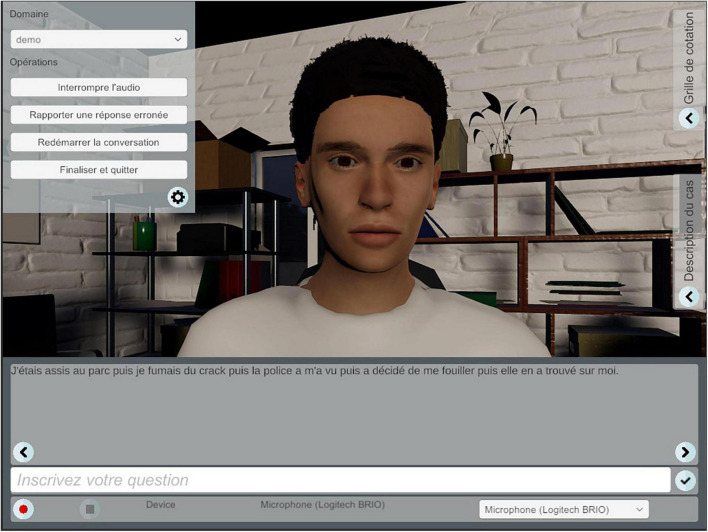
The chatbot program interface.

In this specific study, the chatbot portrays a young adult on probation following a teenage sentence, and the chatbot appeared in a setting that resembled a traditional professional’s office. To provide a realistic experience to users, the scenario (youth response) is based on a real young adult followed in a youth center in Quebec. We conducted interviews and asked him to answer questions generated in a previous data collection. We asked the participant to respond as naturally as possible. The interviews were filmed, and his voice was recorded. Figure 1 shows the chatbot program interface.

### Statistical analysis

The collected data was analyzed using SPSS statistical software version 25 (IBM Corp, 2017). The general characteristics of the participants were analyzed using frequency, percentages, means (M), and standard deviations (SD). Student’s *t*-tests were conducted to compare the means of acceptance and trust in two age and gender group. To investigate the relationship between acceptance, trust and the participant’s characteristics, Pearson’s correlation coefficients were used for continuous variables and means comparisons for categorical variables. Multiple regression analysis was conducted to analyze the factors influencing the subject’s trust and acceptance of the chatbot.

## Measures

Sociodemographic Questionnaire: Participants were asked to complete a standard sociodemographic questionnaire. Sociodemographic information collected included age, gender, ethnicity, relationship status, education, type of graduate program, years in the program, present occupation, and desired occupation.

### AES

To measure the acceptance of the chatbot we used the validated French version of the Acceptability E-scale (AES) (Tariman et al., 2011; Micoulaud-Franchi et al., 2016). This scale is a 6-item scale designed to assess usability (i.e., the perceived ease of using the system or app) and satisfaction (i.e., the perceived enjoyment of the use and usefulness of the system or app). All items were measured on a 1 (very unsatisfied) to 5 (very satisfied) Likert-like scale. Total scores can range from 6 to 30, with a higher score indicating higher acceptance. The internal consistency of this scale ranges between 0.70 and 0.76 (Tariman et al., 2011; Micoulaud-Franchi et al., 2016) which is similar to the internal consistency of 0.79 found in the current study. An example of an item for usability is “How easy was this computer program for you to use?” and an example for satisfaction is “How much did you enjoy using this computer program?” The original version of the AES has been validated with an English-speaking adult population being treated for various forms of cancer (Tariman et al., 2011). The French version of the scale was validated with a sample of 178 French-speaking patients having psychiatric or sleep complaints (Micoulaud-Franchi et al., 2016).

### ETQ

To measure the students’ perceived trust of the chatbot, we used the ECA Trust Questionnaire (ETQ) (Philip et al., 2020). This six-item French questionnaire is designed to assess users’ trust in virtual agents based on two subdimensions: perceived credibility (ability and expertise of the virtual agent) and perceived benevolence (well-intentioned and accurately take the user’s interests into account). All items were measured on a 1 (disagree strongly) to 5 (agree strongly) Likert-like scale. Total scores can range from 0 to 18, with a higher score indicating a more favorable attitude toward the agent. The internal consistency of this scale is 0.71 (Philip et al., 2020), while the internal consistency found in the present study is 0.30. An example of an item for perceived credibility is “Did you feel that the virtual agent was competent?” and an example for benevolence is “Did you feel that the interview with the virtual agent was pleasant?” The scale was validated with a sample of 318 patients suffering from various sleep disorders (Philip et al., 2020).

### Mini-IPIP

To measure participants’ personality traits we used the validated French version of the Mini-International Personality Item Pool (Mini-IPIP) (Donnellan et al., 2006; Laverdière et al., 2020). This 20-item scale is designed to evaluate personality traits according to the Big Five Model. Each of the five factors (neuroticism, extraversion, intellect, agreeableness, and conscientiousness) were assessed with four items, comprising a total scale that included 20 items. All items were measured on a 1 (very inaccurate) to 5 (very accurate) Likert-like scale. The internal consistency of this scale ranged between 0.64 and 0.81 (Donnellan et al., 2006; Laverdière et al., 2020) while the internal consistency found in the present study is 0.59. Example of item include “Have frequent mood swings” (neuroticism), “Talk to a lot of different people at parties” (extraversion), “Have a vivid imagination” (intellect), “Feel others’ emotions” (agreeableness), and “Get chores done right away” (conscientiousness). The Mini-IPIP was validated with a sample of 139 French-Canadian psychology undergraduates (Laverdière et al., 2020).

### Immersive tendencies questionnaire

To measure participants’ immersive tendencies we used the validated French version of Immersive Tendencies Questionnaires (Witmer and Singer, 1998; Robillard et al., 2002). This 18-item scale is designed to assess the degree to which a participant may easily feel immersed and present in virtual environments. All items were measured on a 1 (never) to 7 (often) Likert-like scale. The scale is separated into four separate subscales related to four distinct tendencies of immersion: focus on current activities, involvement in activities, emotions, and tendency to play video games. Total scores can range from 18 to 126, with a higher score indicating more immersive tendencies. The internal consistency of this scale is 0.78 (Robillard et al., 2002) while in the present study, the internal consistency found is 0.69. Example of items includes “Do you easily become deeply involved in movies or TV dramas?” (focus on current activities), “How frequently do you find yourself closely identifying with the characters in a story line?” (involvement in activities), “Do you ever have dreams that are so real that you feel disoriented when you awake?” (emotions) and “How often do you play arcade or video games?” (tendency to play video games). The French version of the scale was validated with a sample of 94 participants who were taking part in a virtual immersion activity (Robillard et al., 2002).

### STAI-Y

To measure participants’ anxiety we used the validated French version of the State-trait Anxiety Inventory (STAI-Y) (Spielberger, 1989; Gauthier and Bouchard, 1993). This 40-item scale is divided into two subscales, which measure state and trait anxiety. The state anxiety scale consists of 20 items (item 1 to item 20) that measure the respondent’s feeling at that moment. The trait anxiety scale consists of 20 items (item 21 to item 40), and this scale measures how the respondent “generally” feels. Each item of the STAI-Y is rated on a scale of 1 (not at all) to 4 (very much so) in terms of intensity for state anxiety (not at all = 1, somewhat = 2, moderately so = 3, very much so = 4) and on a scale of 1 (almost never) to 4 (almost always) in terms of frequency for trait anxiety. Scores range from 20 to 80 per subscale, with a higher score indicating a higher degree of state and/or trait anxiety. The internal consistency of this scale ranges between 0.86 and 0.95 (Spielberger, 1989; Gauthier and Bouchard, 1993) and in the present study the internal consistency is 0.94. State anxiety items include “I am tense” while trait anxiety items include “I worry too much over something that really doesn’t matter.” The STAI-Y’s English and Spanish version were validated with two samples: 38 Spanish-English teachers and teacher assistants and 31 English-education undergraduates from Puerto Rico (Spielberger, 1989). Its French version was validated with a sample of 83 psychology undergraduates from Laval University in Quebec (Gauthier and Bouchard, 1993).

### LSQ-Fa

To measure participants’ learning styles we used the abridged French version of the Learning Style Questionnaire (LSQ; Honey and Mumford, 1982; Fortin et al., 1997). This 48-item questionnaire is designed to assess preference for learning methods. Of the 48 items, there are 12 items for every learning style (active, reflector, theorist, and pragmatist). All items were measured on a 1 (totally disagree) to 7 (strongly agree) Likert-like scale. The total score for each learning style ranges between 12 and 84, with a higher score indicating a higher preference for the learning style. The internal consistency of this scale ranges between 0.86 and 0.95 (Fortin et al., 1997) and in the present study the internal consistency found is 0.85. Active style items include “I like to be the one who talks a lot,” reflector style items include “I am careful not to jump to conclusions too quickly,” theorist style items include “I like to be able to relate my actions to a general principle” and pragmatist style items include “In discussions, I like to get straight to the point.” The French version of the LSQ has been validated with 205 university students in education (Fortin et al., 1997).

### Self-efficacy questionnaire

Based on the available research (Schwarzer and Jerusalem, 1995; Delgadillo et al., 2014; Washburn et al., 2020), we developed a 12-item questionnaire to assess the sense of perceived self-efficacy within the use of the risk assessment tool. All items were measured on a 1 (disagree strongly) to 5 (agree strongly) Likert-like scale. Total scores can range from 12 to 60, with a higher score indicating more self-efficacy within the use of the risk assessment tool. To ensure the internal consistency of the scale, Cronbach’s alpha coefficients were calculated. According to Cronbach’s threshold, analyses showed good results (α = 0.85). An example of items includes “In an interview, I know how to address the different themes included in the YLS/CMI.”

## Results

### Characteristics of the participants

A total of 112 students were analyzed. Participant characteristics are summarized in Table 1. Results show that participants were mostly female (85.7%) between 20 and 25 years old (75.9%). The highest level of education was mostly a college-level diploma (55.4%) and, except for one, all of them were in a criminology program (92.9%), mostly in their second year (79.5%). Participant occupations were mostly full-time student and part-time job (58%).

**TABLE 1:** Sociodemographic characteristics of participants.

Characteristics		
	**(*N* = 112)**	**%**
**Gender**		
Female	96	85.7%
Male	12	10.7%
**Age**		
Under 20	3	2.7%
20–25	85	75.9%
26–30	12	10.7%
31–35	4	3.6%
36–40	2	1.8%
Older than 41	2	1.8%
**Highest level of education**		
Diploma of vocational or college studies	62	55.4%
Certificate/Bachelor’s degree	44	39.3%
Master’s degree	1	0.9%
**Actual level of education**		
First year of bachelor’s degree	4	3.6%
Second year of bachelor’s degree	89	79.5%
Third year of bachelor’s degree	10	8.9%
Master	1	0.9%
**Discipline of actual education**		
Criminology	104	92.9%
Independent studies	1	0.9%
**Current occupation**		
Full-time student	24	21.4%
Part-time student	2	1.8%
Full-time job	2	1.8%
Full-time student and full-time job	9	8.0%
Full-time student and part-time job	65	58.0%
Part-time student and full-time job	4	3.6%
Part-time student and part-time job	2	1.8%

### Acceptance and trust perception with the chatbot

#### Acceptance

As shown in Table 2, results indicate that the overall system acceptance (satisfaction and usability subscales) was rated mostly positively by the participants, with more than half being “satisfied” or “very satisfied” with every item of the scale. Results show that median scores for all the items were 4 (satisfied), which means that half the scores are greater than or equal to “satisfied” and half are lower.

**TABLE 2:** Distribution of satisfaction and usability subscales.

	Score					Descriptive statistics		
**Items**	**Very unsatisfied**	**Unsatisfied**	**Neutral**	**Satisfied**	**Very satisfied**	**M** **(SD)**	**Mdn**	**Min-Max**
**Satisfaction**								
How much did you enjoy using this chatbot?	1 (0.9%)	6 (5.4%)	17 (15.2%)	66 (58.9%)	22 (19.6%)	3.91 (0.80)	4	1–5
How useful was this chatbot to you in assessing the risk of recidivism?	0 (0%)	6 (5.4%)	20 (17.9%)	57 (50.9%)	29 (25.9%)	3.97 (0.81)	4	2–5
How would you rate your overall satisfaction with this chatbot?	2 (1.8%)	8 (7.1%)	30 (26.8%)	62 (55.4%)	10 (8.9%)	3.63 (0.82)	4	1–5
**Usability**								
How easy was this chatbot for you to use?	0 (0%)	12 (10.7%)	21 (18.8%)	68 (60.7%)	11 (9.8%)	3.70 (0.79)	4	2–5
How understandable were the answers provided by the chatbot?	1 (0.9%)	24 (21.4%)	23 (20.5%)	55 (49.1%)	9 (8%)	3.42 (0.95)	4	1–5
How acceptable is the time spent asking questions to this chatbot?	6 (5.4%)	26 (23.2%)	17 (15.2%)	36 (32.1%)	27 (24.1%)	3.46 (1.24)	4	1–5

Concerning satisfaction, results indicate that most participants enjoyed using the chatbot, with 78.5% being “satisfied” or “very satisfied.” Participants also found the chatbot useful for risk assessment training, with 76.8% being either “satisfied” or “very satisfied” and 5.4% being “unsatisfied” and no one being “very unsatisfied.” Overall, participants were mostly satisfied with the chatbot, with 64.3% being “satisfied” or “very satisfied.” As for usability, results indicate that participants mostly found the chatbot easy to use, with over 70% being “satisfied” or “very satisfied” and 10.7% being “unsatisfied” and no one being “very unsatisfied.” Results show that 57.1% of participants were “satisfied” or “very satisfied” with the answers provided by the chatbot during the exercise, while 22.3% were “unsatisfied” or “very unsatisfied.” More than half of the participants also found that the time spent asking questions to the chatbot was acceptable, with 56.2% being “satisfied” or “very satisfied” and 28.6% were “unsatisfied” or “very unsatisfied.”

According to comments made in the qualitative section of the questionnaire, the lower usability level in this study is likely due to technical issues that some participants experienced during the study. The first technical issue reported by participants is that the chatbot software was too resource intensive for their computer. For example, one participant stated that “The biggest difficulty I encountered was on the computer side. Indeed, after 5 min of use, my computer was overheating, so I had to quit and come back each time” [author’s translation]. The second technical issue also reported by participants is that during the exercise they had to restart the conversation with the chatbot several times. One participant stated that:

“After a few hours of consecutive use, the chatbot simply stopped answering my questions, even if I reset the conversation. So, I had to quit the application and restart it so that it would start answering again. It wasn’t a big problem and didn’t bother me much, but I just wanted to share it with you” [author’s translation].

#### Trust

As shown in Table 3, results indicate that the overall system trust (benevolence and credibility subscales) was rated more positively than negatively by the participants. Except for the item “Did you feel that your questions were correctly understood by the chatbot,” more than half responded that they were either neutral or agreed with all items. Results show that median scores for all the items, except for the one named above, were 3 (neutral), which means that half the scores are greater than or equal to “neutral” and half are lower.

**TABLE 3:** Distribution of benevolence and credibility subscales.

	Score					Descriptive statistics		
**Items**	**Strongly disagree**	**Disagree**	**Neutral**	**Agree**	**Strongly Agree**	**M** **(SD)**	**Mdn**	**Min-Max**
**Benevolence**								
Did you feel that your questions were correctly understood by the chatbot?	6 (5.4%)	61 (54.5%)	18 (16.1%)	1 (0.9%)	26 (23.2%)	2.82 (1.30)	2	1–5
Did you feel that the answers provide by the chatbot were clear?	2 (1.8%)	21 (18.8%)	60 (53.6%)	6 (5.4%)	23 (20.5%)	3.24 (1.04)	3	1–5
Did you feel that the interview with the chatbot was pleasant?	2 (1.8%)	15 (13.4%)	54 (48.2%)	14 (12.5%)	27 (24.1%)	3.44 (1.05)	3	1–5
**Credibility**								
The chatbot should be integrated into training practices?	1 (0.9%)	4 (3.6%)	53 (47.3%)	39 (34.8%)	15 (13.4%)	3.56 (0.80)	3	1–5
The chatbot should obligatory be used in training?	5 (4.5%)	19 (17%)	33 (29.5%)	19 (17.0%)	36 (32.1%)	3.55 (1.23)	3	1–5
Did you feel that the chatbot was credible?	2 (1.8%)	9 (8%)	60 (53.6%)	18 (16.1%)	23 (20.5%)	3.46 (0.98)	3	1–5

Concerning benevolence, when asked if their questions were correctly understood by the chatbot, 59.9% of participants disagreed with this statement (disagree or strongly disagree). As for the answers provided by the chatbot, participants most often neither agreed nor disagreed (53.6%) with the clarity of the answers provided by the chatbot. Results also show that 48.2% of participants found the interview with the chatbot neither pleasant nor unpleasant, while 36.6% found that it was pleasant. As for credibility, almost half the participants agreed with the integration of the chatbot into training practices and with the mandatory integration at 48.2% and 49.1%, respectively. As for the credibility of the chatbot, 53.6% of participants neither agreed nor disagreed with it.

According to comments made in the qualitative section of the questionnaire, the lower trust levels in this study are likely due to logistic issues that participants experienced during the study. The first and main logistical issue reported by participants is that the chatbot did not understand several of their questions. One participant states that “The difficulty I encountered that stood out the most in my use of the chatbot was the fact that there were so many questions that led to an answer like ‘I don’t understand the question”’ [author’s translation]. Participants also indicate that because of this issue, the session was time-consuming. For example, one student said, “I felt like I spent more time trying to write questions that he understood rather than doing the scoring [of the YLS/CMI] itself” [author’s translation]. Because of this, multiple participants also experienced frustration and anxiety. One participant said, “it can be frustrating to ask questions that you think are necessary for your rating and the chatbot just doesn’t have the answer, no matter how you ask it” [author’s translation]. Another participant stated that “[…] the fact that we were evaluated on the exercise made the whole thing very stressful and increased the frustration of the normal misunderstanding of the chatbot when faced with certain questions” [author’s translation].

### Factors associated with acceptance and trust

#### Age and gender

As presented in Tables 4, 5, results showed no significant relationship between the satisfaction, usability, benevolence, credibility subscales and age or gender.

**TABLE 4:** Differences in acceptance and trust subscales scores between younger and older adult.

	Under 30 years old		Over 30 years old		*t*	*p*	Cohen’s *d*
	***M***	***SD***	***M***	***SD***			
Acceptance–Satisfaction	3.814	0.682	3.833	0.690	−0.740	0.941	0.027
Acceptance–Usability	3.501	0.706	3.791	0.754	−1.111	0.269	0.397
Trust–Benevolence	3.168	0.639	3.166	0.816	0.007	0.994	0.002
Trust–Credibility	3.532	0.622	3.083	0.849	1.907	0.059	0.603

**TABLE 5:** Differences in acceptance and trust subscales scores between female and male.

	Female		Male		*t*	*p*	Cohen’s *d*
	***M***	***SD***	***M***	***SD***			
Acceptance–Satisfaction	3.854	0.652	3.484	0.848	−1.723	0.088	0.489
Acceptance–Usability	3.559	0.684	3.212	0.885	−1.543	0.126	0.438
Trust–Benevolence	3.194	0.629	2.939	0.800	−1.236	0.219	0.354
Trust–Credibility	3.524	0.631	3.272	0.771	−1.222	0.224	0.357

#### Acceptance

**FIGURE 2:**
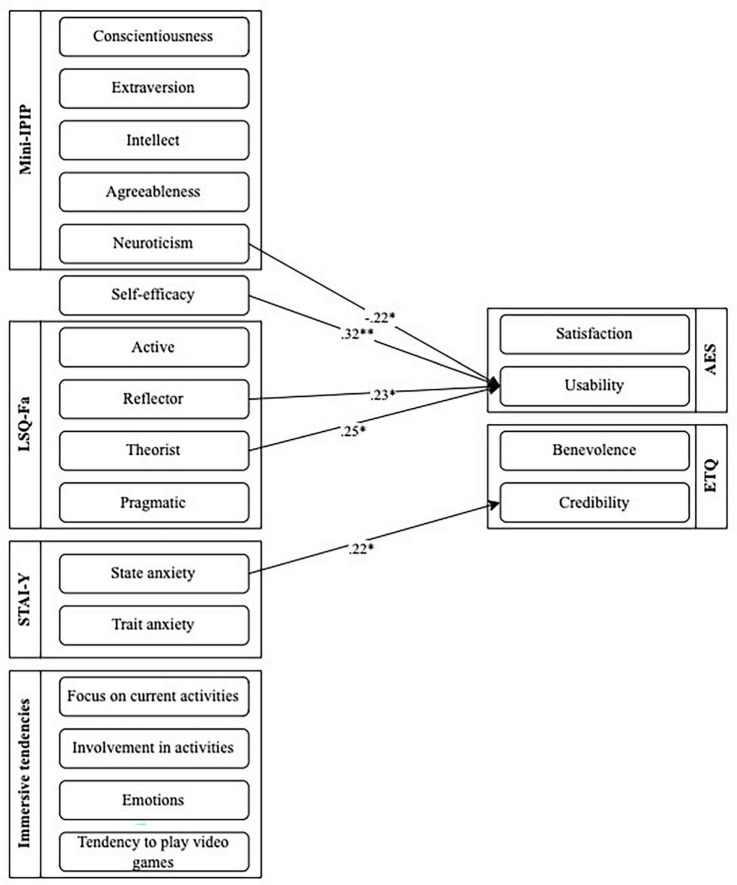
Correlation coefficients between user-related factors and acceptance and trust of the chatbot.

As shown in Table 6, a series of Pearson’s correlations were conducted to determine if there was any significant relationship between acceptance and diverse factors identified in research. Regarding satisfaction subscale of the AES, results showed no significant relationship with any variables. As for the usability subscale of the AES, results showed moderate positive correlations with the theorist learning style of the LSQ-Fa (*r* = 0.25; *p* < 0.05) and the reflector learning style of the LSQ-Fa (*r* = 0.23; *p* = 0.05). Results also show a moderate negative correlation with the neuroticism dimension of the Mini-IPIP (*r* = −0.22; *p* < 0.05). Results show high positive correlations with the self-efficacy scale (*r* = 0.32; *p* < 0.01). Figure 2 presents the relationship between acceptance and these variables.

**TABLE 6:** Correlations between acceptance, trust, and independent variables.

	Acceptance–Satisfaction	Acceptance–Usability	Trust–Benevolence	Trust– Credibility
**Mini-International Personality Item Pool (Mini-IPIP)**				
Neuroticism	−0.00	−0.22*	−0.05	0.14
Extraversion	0.05	−0.01	0.11	0.08
Intellect	−0.03	−0.04	−0.08	−0.12
Agreeableness	0.07	0.02	−0.06	−0.09
Conscientiousness	0.18	0.10	0.01	0.06
**Immersive Tendencies Questionnaire**				
Focus on current activities	0.05	−0.07	−0.03	−0.05
Involvement in activities	0.06	−0.06	−0.10	−0.14
Emotions	0.08	0.19	−0.04	−0.01
Tendency to play video games	−0.05	−0.08	−0.11	−0.01
**State-trait Anxiety Inventory (STAI-Y)**				
Trait anxiety	−0.10	−0.18	0.00	0.15
State anxiety	0.03	−0.04	0.09	0.22*
**Learning Style Questionnaire-Fa (LSQ-Fa)**				
Active	0.10	−0.02	0.03	−0.02
Reflector	−0.04	0.23*	0.06	0.07
Theorist	0.09	0.25*	0.03	0.14
Pragmatist	0.07	0.14	0.07	−0.02
Self-efficacy questionnaire	0.19	0.32**	−0.02	−0.06

*p < 0.05; **p < 0.01.

#### Trust

As shown in Table 6, a series of Pearson’s correlations were also conducted to determine if there was any significant relationship between trust and diverse factors identified in research. Concerning the benevolence subscale of the ETQ, results showed no significant relationship with any variables. Regarding the credibility subscale of the ETQ, results showed a moderate positive correlation between the state anxiety dimension of the STAI-Y (*r* = 0.22; *p* < 0.05). Figure 2 also presents the relationship between trust and these variables.

**FIGURE 3:**
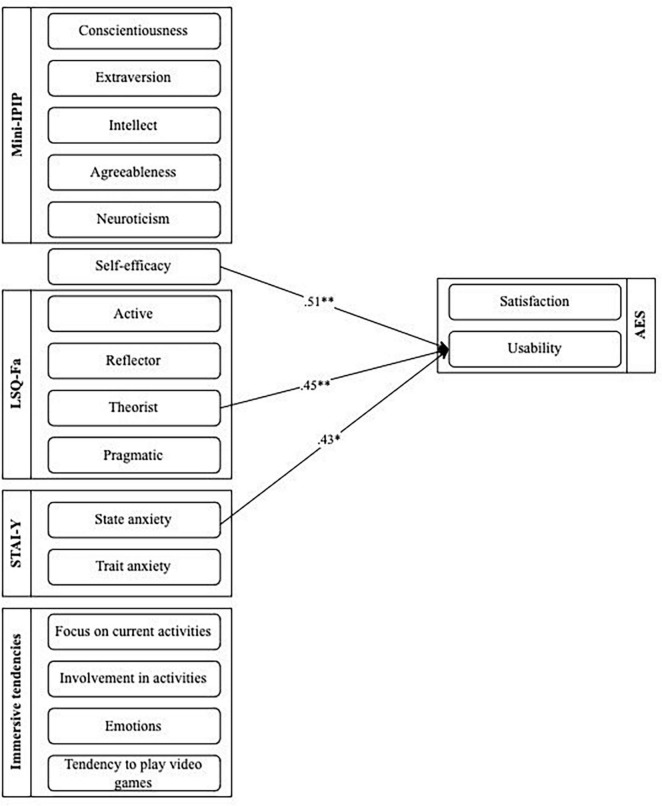
Linear regression coefficients between user-related and factors and perception of usability.

As shown in Table 7, multivariate analyses were conducted. A significant regression equation was found only for the usability subscale [*F*(16.24) = 1.951, *p* < 0.005], with an *R*^2^ of 0.33. Results show significant relationships between usability and state anxiety (*b* = 0.43; *p* < 0.05), self-efficacy (*b* = 0.51; *p* < 0.01), and the theorist learning style (*b* = 0.45; *p* < 0.05). Models predicting satisfaction, benevolence and credibility subscales were not significant (see Supplementary Tables 1–3). Figure 3 presents the relationship between usability and these variables.

**TABLE 7:** Linear regressions of factors associated with usability with the chatbot.

Independent variables	Coefficient	S.E.	Beta	*T*
**Mini-International Personality Item Pool (Mini-IPIP)**				
Neuroticism	−0.11	0.13	−0.14	−0.85
Extraversion	−0.05	0.13	−0.07	−0.42
Intellect	−0.02	0.13	−0.02	−0.18
Agreeableness	0.27	0.15	0.22	1.78
Conscientiousness	−0.06	0.11	−0.07	−0.52
**Immersive Tendencies Questionnaire**				
Focus on current activities	−0.03	0.09	−0.05	−0.38
Involvement in activities	−0.09	0.10	−0.11	−0.90
Emotions	0.13	0.10	0.17	1.30
Tendency to play video games	0.03	0.06	0.05	0.43
**State-trait Anxiety Inventory (STAI-Y)**				
Trait anxiety	−0.37	0.29	−0.25	−1.28
State anxiety	0.43*	0.20	0.34	2.12
**Learning Style Questionnaire-Fa (LSQ-Fa)**				
Active	−0.02	0.16	−0.02	−0.12
Reflector	−0.15	0.15	−0.15	−0.99
Theorist	0.45**	0.17	0.42	2.71
Pragmatist	−0.01	0.16	−0.01	−0.07
Self-efficacy questionnaire	0.51**	0.17	0.36	2.98

*p < 0.05; **p < 0.01.

## Discussion

The objective of this study was twofold: (1) to examine the perceived acceptance and trust in a risk assessment training chatbot developed to assess risk and needs of juvenile offenders, and (2) to examine the factors influencing students’ perception of acceptance and trust. These findings are very encouraging and suggest that the chatbot could be an effective educational method.

### Acceptance and trust perception

Taken together the results of the present study show satisfactory level of acceptance and trust in chatbot. Overall, except for few technical and logistical limitations, the chatbot was functional and well-appreciated by participants. These findings are consistent with other studies that show satisfactory levels of acceptance involving chatbots (Philip et al., 2020; Richardson et al., 2021). In prior works, most respondents appeared to find chatbots acceptable and usable, but they also mentioned some technical issues that affected their experience. As pointed out by Richardson et al. (2021), even if most participants found the virtual software usable, all participants suggested some form of technological amendment to improve the user experience. As for trust, few studies have looked at the notion of trust, and available studies have focused on the notion of trust in a customer service context or in clinical interviews with patients (Philip et al., 2020).

### Factors associated with acceptance and trust

Several factors associated with acceptance and trust have been identified in this study, but mostly with the usability dimension. Results show that learning style has an influence on chatbot acceptance. More precisely, results show that the theorist learning style has an influence on usability. Reflective and analytical people who like to understand the theories behind actions seem to find the chatbot easier to use. As theorists enjoy following models and reading up on facts to better engage in the learning process, those people may have taken the time to clearly understand the functioning of the chatbot and its features.

Results also demonstrate that self-efficacy has an influence on chatbot usability. People who have a higher sense of efficacy within the use of the risk assessment tool seem to find the chatbot easier to use. This result is consistent with previous research which suggests that self-efficacy has a potential impact on acceptance and trust (Agarwal et al., 2000; Czaja et al., 2006). Participants with higher belief in their own capacity to achieve the risk assessment’s exercise found the chatbot easier to use. As risk assessment is a complex task with important consequence on individual liberties and public security, future professionals must feel competent in their risk assessment. There is also some evidence supporting that computer self-efficacy (one’s belief about his ability to perform a specific task using a computer) (Compeau and Higgins, 1995) has been shown to be a strong determinant of perceived ease of use before firsthand experience (Agarwal et al., 2000; Venkatesh, 2000). It will be interesting to integrate this specific type of self-efficacy in future work.

Results point out that state anxiety has an influence on chatbot usability. People who present situational anxiety like unpleasant feelings of tension and apprehension seem to find the chatbot easier to use. This result is consistent with the studies on trust and on computer anxiety. Indeed, as suggested by Müller et al. (2019), people who experience more anxiety tend to present a higher ability to trust chatbots. As credibility also measures the relevance of a chatbot in risk assessment training in this study, this result may suggest that the chatbot can be an effective training method for anxious students. Anxious people can find the chatbot credible but also easier to use because the chatbot allows students to put into practice the theory learned in a much more concrete way, which can reduce the level of anxiety during exercise. This allows students to practice their interview skills without the time constraints and pressure/stress of the task in the practical setting. In addition, chatbot programs offer students a space to safely try new approaches and new techniques without any consequences for real clients. We found consistent, but not statistically significant, differences between males and females for acceptance, usability, benevolence, and credibility. The results show slight differences between men and women, i.e., women seem to find the chatbot more acceptable and reliable than men. The results of this study are negatively impacted by the small sample size because small sample sizes significantly decrease statistical power and the flexibility of detecting any type of effect size (Heidel, 2016). In addition, there are few males in the sample since there were less men than women in the mandatory course on risk assessment. Future research should include a heterogeneous sample. The findings in the current study are consistent with recent research on technology acceptance.

We also found no significant differences between age for acceptance, usability, benevolence, and credibility. Some research highlighted differences between older and younger adults, whereas others did not (Grimes et al., 2010; Mitzner et al., 2010; McLean and Osei-Frimpong, 2019; Følstad and Brandtzaeg, 2020). Our result could be explained by age-homogeneous composition of the sample. Since most participants were between 20 and 25 years old, it is more difficult to compare groups.

## Strengths and limitations

This study has several strengths and implications. First, to our knowledge, there are no chatbots with virtual avatar available that are useful for training professionals who assess the risk of recidivism of offenders. This is the first study to examine the perceived acceptance and trust in a risk assessment training chatbot. Considering the consequences that such assessments have on individual liberties and public security, developing effective and realistic training methods is warranted. Second, this study has also highlighted some factors that are associated with the acceptance and trust of a chatbot, such as self-efficacy, learning style and anxiety. This study provides a better understanding of the factors that facilitate user acceptance and trust of a chatbot, and a solution to the modifications needed for successful adoption. Future studies should examine those factors because such investigations may provide more comprehensive information regarding how to successfully integrate a chatbot into training programs.

This study also has several limitations. The first one is that the chatbot exercise was conducted as a mandatory exercise in a risk assessment course. Since this was a practical examination, it is possible that students answered and reacted differently. For example, they may have experienced more stress knowing that they were going to be graded following the exercise. The second limit is that the sample was homogeneous (i.e., age and gender), which makes it difficult to compare the groups. In addition, the generalizability of this study is limited by the lack of diversity in the sample. It would be interesting to examine chatbot’s acceptance and trust with working professionals, who do not feel pressure to succeed and who represent a more heterogeneous group. The number and length of online questionnaires is the third limit of this study. Prior work has found the length of questionnaires to affect response rate (Sahlqvist et al., 2011). The response rate may therefore have been affected by the number and length of questionnaires in this study. Finally, the reliability of the ECA Trust Questionnaire is also a limit, as it shows an extremely low internal consistency. Such a difference between the original reliability and our findings can be explained by the different population composing the different samples. The ECA Trust Questionnaire may not be an appropriate scale to use with students or professionals working in the risk assessment field. If this study was to be replicated, another measurement scale should be developed to evaluate trust.

## Conclusion

The objective of the current study was to examine the perceived acceptance and trust in a risk assessment training chatbot developed to assess risk and needs of juvenile offenders and the factors influencing acceptance and trust. Results show a high level of acceptance in the chatbot. Participants were satisfied with their experience with the chatbot. Most users found the chatbot easy to use, even if they noted some technical issues, such as resource intensive software and conversation problems. As for trust in the chatbot, results show a satisfactory level. Participants found that the chatbot was benevolent, but numerous participants reported that the chatbot did not understand nor answered several of their questions. As for credibility, participants found the chatbot credible. They mentioned being in favor of integration into practice, but perhaps not as a mandatory evaluation.

Furthermore, results also suggest that acceptance and trust do not only depend on the design of the chatbot software, but may also vary depending on the characteristics of the user. Results suggested that self-efficacy, state anxiety and learning styles have an influence on the acceptance and trust of a chatbot, and especially on usability. Analytical individuals and anxious individuals seem to find the chatbot easier to use. Those who found the chatbot easier to use had higher belief in their own capacity to achieve the risk assessment’s exercise.

As trust and acceptance play a vital role in determining technology success, these results are encouraging. Future studies are required to explore how several factors influence acceptance and trust in a risk assessment training chatbot. However, as reported by participants, some improvements need to be done prior to that. Since there are no such chatbots available for training professionals working in the fields of clinical psychology, psychiatry social work and criminology, these results are important as they tell us about the limitations of chatbots and the modifications that are needed.

## Data availability statement

The original contributions presented in this study are included in the article/Supplementary material, further inquiries can be directed to the corresponding author.

## Ethics statement

The studies involving human participants were reviewed and approved by Comité d’éthique de la recherche–Société et culture at the University of Montreal (#CERSC-2022-024-D) and Comité d’éthique de la recherche–Jeunes en difficulté (#MP-CER-JD-20-19). The patients/participants provided their written informed consent to participate in this study.

## Author contributions

A-PR and J-PG contributed to the conception and design of the study. A-PR organized the database, performed the statistical analysis, and wrote the manuscript. All authors contributed to the manuscript revision and read and approved the submitted version.
