# Supplementary material for: Factors influencing acceptance and trust of chatbots in juvenile offenders’ risk assessment training
Source: Front Psychol. 2023 Jun 16;14:1184016. doi: 10.3389/fpsyg.2023.1184016 (PMC10312240; doi:10.3389/fpsyg.2023.1184016)
Supplement: Supplementary file 1 [file Data_Sheet_1.docx]

Supplementary Material

Factors influencing acceptance and trust of chatbots in juvenile offenders’ risk assessment training

**Ann-Pierre Raiche*, Léanne Dauphinais, Manon Duval, Gino De Luca, David Rivest-Hénault, Thomas Vaughan, Catherine Proulx, Jean-Pierre Guay**

Ann-Pierre Raiche*

[Ann-pierre.raiche@umontreal.ca](mailto:Ann-pierre.raiche@umontreal.ca)

# Supplementary Tables

| Independent variables | Coefficient | S.E. | Beta | T |
| --- | --- | --- | --- | --- |
| Mini-International Personality Item Pool (Mini-IPIP) |  |  |  |  |
| Neuroticism | .11 | .13 | .02 | .09 |
| Extraversion | -.04 | .12 | -.06 | -.35 |
| Intellect | -.03 | .12 | -.04 | -.27 |
| Agreeableness | .15 | .15 | .13 | .97 |
| Conscientiousness | .25 | .11 | .32 | 2.30 |
| Immersive Tendencies Questionnaire |  |  |  |  |
| Focus on current activities | .01 | .09 | .01 | .07 |
| Involvement in activities | .01 | .09 | .01 | .07 |
| Emotions | .16 | .10 | .23 | 1.60 |
| Tendency to play video games | -.04 | .06 | -.09 | -.72 |
| State-trait Anxiety Inventory (STAI-Y) |  |  |  |  |
| Trait anxiety | -.13 | .28 | -.10 | -.47 |
| State anxiety | .30 | .20 | .26 | 1.55 |
| Learning Style Questionnaire-Fa (LSQ-Fa) |  |  |  |  |
| Active | .09 | .16 | .11 | .60 |
| Reflector | -.27 | .14 | -.30 | -1.86 |
| Theorist | .14 | .16 | .15 | .90 |
| Pragmatist | .05 | .16 | .05 | .31 |
| Self-efficacy questionnaire | .26 | .17 | .20 | 1.55 |

**Table 1.** Linear regressions of factors associated with satisfaction with the chatbot

| Independent variables | Coefficient | S.E. | Beta | T |
| --- | --- | --- | --- | --- |
| Mini-International Personality Item Pool (Mini-IPIP) |  |  |  |  |
| Neuroticism | -.06 | .13 | -.09 | -.46 |
| Extraversion | .19 | .12 | .28 | 1.55 |
| Intellect | .02 | .12 | .03 | .20 |
| Agreeableness | -.02 | .15 | -.02 | -.15 |
| Conscientiousness | -.06 | .11 | -.08 | -.50 |
| Immersive Tendencies Questionnaire |  |  |  |  |
| Focus on current activities | -.02 | .09 | -.03 | -.21 |
| Involvement in activities | -.00 | .09 | -.00 | -.02 |
| Emotions | -.07 | .10 | -.10 | -.68 |
| Tendency to play video games | .05 | .06 | .10 | .75 |
| State-trait Anxiety Inventory (STAI-Y) |  |  |  |  |
| Trait anxiety | -.28 | .28 | -.23 | -1.02 |
| State anxiety | .19 | .19 | .18 | .99 |
| Learning Style Questionnaire-Fa (LSQ-Fa) |  |  |  |  |
| Active | -.15 | .16 | -.19 | -.92 |
| Reflector | .11 | .14 | .14 | .80 |
| Theorist | .12 | .16 | .14 | .78 |
| Pragmatist | .06 | .16 | .06 | .36 |
| Self-efficacy questionnaire | -.13 | .17 | -.11 | -.79 |

**Table 2.** Linear regressions of factors associated with benevolence with the chatbot

| Independent variables | Coefficient | S.E. | Beta | T |
| --- | --- | --- | --- | --- |
| Mini-International Personality Item Pool (Mini-IPIP) |  |  |  |  |
| Neuroticism | .14 | .14 | .19 | 1.04 |
| Extraversion | .05 | .13 | .06 | .34 |
| Intellect | -0.6 | .13 | -.06 | -.46 |
| Agreeableness | -.18 | .16 | -.15 | -1.12 |
| Conscientiousness | -.02 | .12 | -.03 | -.19 |
| Immersive Tendencies Questionnaire |  |  |  |  |
| Focus on current activities | -.03 | .09 | -.04 | -.29 |
| Involvement in activities | -.13 | .10 | -.18 | -1.29 |
| Emotions | .07 | .11 | .10 | .65 |
| Tendency to play video games | -.04 | .07 | -.09 | -.67 |
| State-trait Anxiety Inventory (STAI-Y) |  |  |  |  |
| Trait anxiety | -.34 | .30 | -.24 | -1.13 |
| State anxiety | .27 | .21 | .23 | 1.29 |
| Learning Style Questionnaire-Fa (LSQ-Fa) |  |  |  |  |
| Active | .02 | .17 | .02 | .11 |
| Reflector | -.06 | .15 | -.06 | -.36 |
| Theorist | .33 | .17 | .33 | 1.94 |
| Pragmatist | -.14 | .17 | -.14 | -.82 |
| Self-efficacy questionnaire | .09 | .18 | .07 | .48 |

**Table 3.** Linear regressions of factors associated with credibility with the chatbot
